# Near-surface termination of upward-propagating strike-slip ruptures on the Yangsan Fault, Korea

**DOI:** 10.1038/s41598-023-37055-7

**Published:** 2023-06-19

**Authors:** Youngbeom Cheon, Chang-Min Kim, Jin-Hyuck Choi, Sangmin Ha, Seongjun Lee, Taehyung Kim, Hee-Cheol Kang, Moon Son

**Affiliations:** 1 Active Tectonics Research Center, Korea Institute of Geoscience and Mineral Resources, Daejeon, 34132 South Korea; 2 Department of Geological Sciences, Pusan National University, Busan, 46241 South Korea; 3 Institute of Geohazard Research, Pusan National University, Busan, 46241 South Korea

**Keywords:** Geodynamics, Geology

## Abstract

We present a new example of the termination of strike-slip paleoearthquake ruptures in near-surface regions on the Yangsan Fault, Korea, based on multi-scale structural observations. Paleoearthquake ruptures occur mostly along the boundary between the inherited fault core and damage zone (N10–20°E/> 75°SE). The ruptures propagated upward to the shallow subsurface along a < 3-cm-wide specific slip zone with dextral-slip sense, along which the deformation mechanism is characterized mainly by granular flow in near-surface region. The ruptures either reach the surface or are terminated in unconsolidated sediment below the surface. In the latter case, the rupture splays show westward bifurcation, and their geometry and kinematics show a change to NNW-strike with low-angle dip and dextral-reverse oblique-slip sense in the strata. We suggest that the upward termination of the contractional strike-slip ruptures is controlled by the inherited fault geometry that is unfavorable with respect to the stress field (ENE–WSW σ_Hmax_) at basement depths in terms of movement on the fault, and the lack of extension of the fault into shallow subsurface; a depth-dependent change in stress from σ_Hmax_ > σ_v_ > σ_Hmin_ to σ_Hmax_ > σ_Hmin_ > σ_v_ at depth of a ~ 200 m; and the physical properties of unconsolidated sediment, which have low inter-granular cohesion, resulting in distributed deformation.

## Introduction

Dynamic earthquake ruptures mainly propagate on inherited faults and show a wide range of deformation styles along and across the fault-strike at and near the ground surface^1,2^. These rupture processes and resultant deformation in shallow subsurface (< 100 m depth) are controlled by various geological and tectonic factors, such as (1) the geometry (or inherited complexity), internal architecture, and the mechanical properties of fault rocks in the fault zone^3–6^; (2) type and thickness of surrounding geological materials (from seismogenic crust to the overlying unconsolidated strata)^7–9^; (3) variations in topography at the surface and lithology (or elastic properties) at depth^10,11^; (4) imposed stress conditions^3,12^; and (5) hypocenter depth and magnitude of the earthquake event^13^. Especially, many reverse and strike-slip ruptures show a sudden reduction of relative seismic velocity at shallow depths^14–16^ and display termination characteristics, such as upward ruptures that terminate below the surface^17^, flower-like damage zones that occur in shallow regions^1,18,19^, slip deficit at shallow levels (i.e., the surface slip is systematically less than slip at depth)^20–22^, and distributed off-fault deformation in the surface^6,23^ at the time of the earthquake.

**Figure 1:**
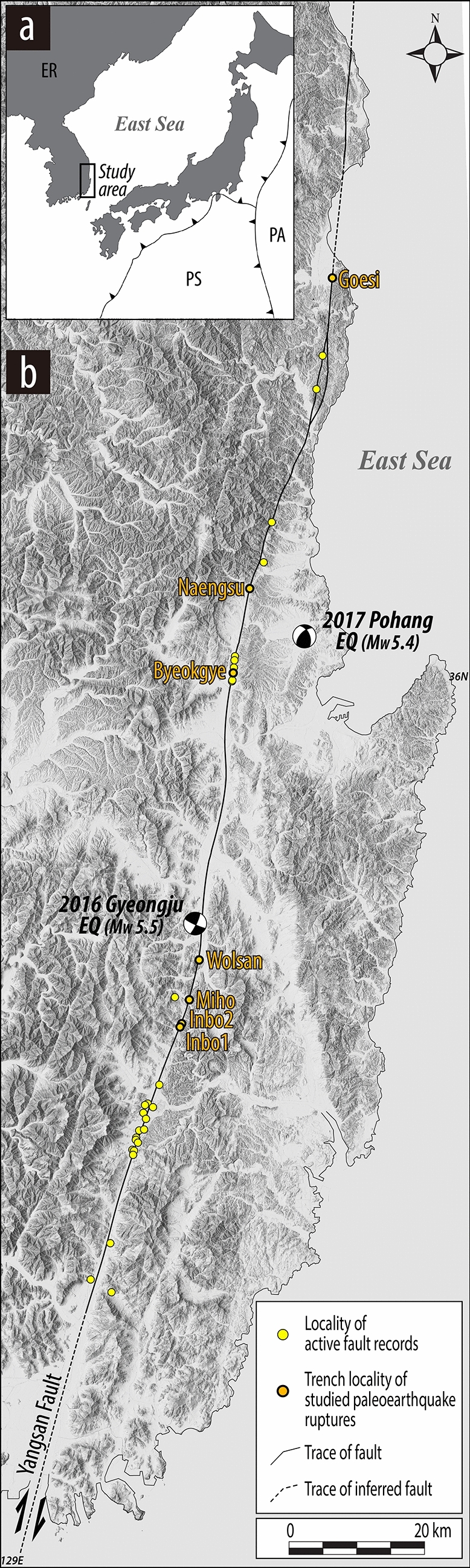
(**a**) Map of the present-day tectonic configuration in East Asia. PA: Pacific Plate, PS: Philippine Sea Plate, ER: Eurasia Plate. (**b**) Map of the Yangsan Fault and locations of Quaternary faulting observations along the fault. A digital elevation model was generated from digitizing topographic maps (1:25,000) from Korean National Spatial Data Infrastructure Portal (http://www.nsdi.go.kr/lxportal/) by using QGIS 3.28.5.

In this study, we introduce a new example of near-surface upward rupture termination during strike-slip paleoearthquakes along the Yangsan Fault on the Korean Peninsula. The Yangsan Fault is a long-lived intraplate fault, as inferred from outcrop- to map-scale structural observations (Fig. 1). It is one of the active seismogenic faults in the Korean Peninsula and was the causative fault of the 2016 Gyeongju Earthquake (M_w_ 5.5), the largest instrumental earthquake recorded in South Korea (Fig. 1). Since this earthquake, scientific and social–economic interests have motivated seismic and paleoseismic studies of the fault, focusing on its recent and future movement and resultant earthquakes. During this study, we searched for geologic evidence of paleoearthquakes and discovered late Quaternary surface ruptures at several excavation sites in the fault zone, where the weakly developed geomorphic features of surface ruptures have remained. We conducted comprehensive structural observations at seven previously excavated trench localities (Fig. 1 and Table 1), including new microstructural data for rupture splays obtained at two previous trenches (Inbo1 and Inbo2 sites in Table 1). In this paper, we organize and present these observations, and then characterize rupture splays that have propagated upward to the shallow subsurface. We highlight the inhibition processes acting on the upward propagation of ruptures, which show a westward bifurcation and change in geometry and kinematics in unconsolidated sediments near the surface. In this paper, we also refer to the results of paleoseismic (e.g., rupture timing and location) and geomorphic characteristics of the Yangsan Fault as reported in our companion papers (Table 1).

**Table 1:** Information on paleoearthquake ruptures and their termination features close to the paleo surfaces at each trench locality from north to south along the Yangsan Fault zone.

Site name	Location (latitude, longitude)	Main core or subsidiary fault	General rupture attitude (strike/dip)	Kinematics	Rupture termination features	References
Geosi	36.53725, 129.41630	Main core	N20°W/< 60°NE	RL + R	No clear evidence due to erosion by artificial deposit	Ko et al.^24^
Naengsu	36.08909, 129.25871	Main core	N15°E/< 42°SE	RL	No clear evidence due to erosion by artificial deposit	Kim et al.^25^
Byeokgye	36.06908, 129.25552	Main core	N28°E/< 86°SE	RL + R	Termination in unconsolidated sediment with changes in geometry and kinematics	Song et al.^26^
Wolsan	35.72288, 129.19450	Main core	N20°E/~ 85°SE	RL	Surface rupture forming fissure filling	Kim et al.^27^
Miho	35.67689, 129.17873	Main core	N27°E/< 75°SE	RL + R	Surface rupture forming colluvial wedge	Kim et al.^27^
Inbo2	35.64764, 129.16640	Subsidiary fault	N05°E/< 67°SE	RL + R	Earthquake horizon (the boundary between fully offset strata by rupture and subsequent overlying strata)	Kim et al.^27^
Inbo1	35.64263, 129.16437	Subsidiary fault	N17°E/< 75°SE	RL + R	Termination in unconsolidated sediment with changes in geometry and kinematics	Cheon et al.^28^

## Background

The Korean Peninsula is situated in the intraplate region of the eastern part of the Eurasian plate, more than 500 km west of the convergent plate boundary between the Eurasia and Pacific plates (see inset of Fig. 1). Because of the small number of damaging instrumental earthquakes recorded before the two recent moderate earthquakes (the 2016 Gyeongju and 2017 Pohang earthquakes in SE Korea; Fig. 1b), the peninsula had long been regarded as a seismologically stable area compared with neighboring countries. The neotectonic regime and stress field in and around the peninsula are controlled by the combined effect of the subducting Pacific and Philippine Sea oceanic plates beneath the Eurasian plate (see inset of Fig. 1) and the far-field stress transmitted by the northward moving Indian plate. The neotectoic setting of the peninsula is known to have been initiated at 5–3.5 Ma (early Pliocene)^29,30^. Comprehensive data obtained from earthquake focal mechanisms, in situ stress measurements (hydrofracturing/overcoring methods), and Quaternary fault slips in the onshore area of the peninsula reveal an ENE–WSW-oriented maximum horizontal stress (σ_Hmax_)^30^. Significantly, the general stress field is regarded as a strike-slip regime (σ_2_ is vertical), as inferred from focal mechanism data^31–33^.

The Yangsan Fault in SE Korea strikes NNE–SSW, dips to the east at a high angle (> 75°), and can be traced for a distance of ~ 200 km on land with a fault core measuring several decameters in width. It transects mainly Mesozoic and Cenozoic sedimentary and igneous rocks, exhibiting dextral kinematics^34,35^. The internal architecture of fault core shows a major single core as well as multiple cores characterized by anastomosing core strands along the entire fault trace^34–36^. This fault has evolved as a crustal-scale mature structure since at least the Late Cretaceous and has undergone multiple stages of deformation/movement due to long-term variation in the regional tectonic environments: (1) Late Cretaceous sinistral-slip faulting; (2) late Paleogene intense dextral-slip faulting; (3) middle Miocene weak sinistral-slip faulting; and (4) local Quaternary dextral-slip faulting^34,35^. The most intense deformation is dextral movement exhibiting 20–35 km of horizontal offset^37–39^ that occurred during the late Paleogene^34^. Historical and paleoseismic studies have shown that some sections (or segments) of the fault have undergone major slip events, giving rise to strong earthquakes and surface ruptures under the modern neotectonic regime^24–28,40,41^. Although noticeable geomorphic features of surface rupture are rare because of the high erosion rates and thick cover of recent sediments, the records of surface ruptures identified at each studied trench wall indicate several rupture events at different times along some fault sections during the late Quaternary^24–27^.

## Data and methods

Multi-scale comprehensive structural observations of paleoearthquake (near-)surface ruptures were made at recently constructed trench localities along the Yangsan Fault (Fig. 1 and Table 1). To understand outcrop-scale structural features, such as distribution, geometry, and kinematics of the rupture splays, we carried out detailed structural mappings on the seven exposed trench walls that were perpendicular to the NNE–SSW-striking fault trace. And then, for revealing the deformation mechanism of the rupture splays in the unconsolidated sediments, we conducted microstructural observations at two sites (Inbo1 and Inbo2 sites) using oriented rock-slabs and thin-sections. We sampled two different contact types of slip zones: (1) an unconsolidated sediment–old fault rock contact (a boundary between the gravel and mature fault gouge at Inbo2 site) and (2) an unconsolidated sediment–sediment contact (a boundary between clay and sand at Inbo1 site). Some limitations were encountered in identifying the above-mentioned two types of slip-zone contact at the one trench-site locality due to exposure conditions of the ruptures. We therefore selected samples from these two nearby sites, which are located on a surface rupture trace, for microscopic observation (Table 1). Oriented hand-specimen samples taken from unconsolidated sediments and clay-rich fault rocks were dried at room temperature for a week and soaked with a low-viscosity epoxy in a vacuumed desiccator^35^. We used low-viscosity oil (electronic discharge machining fluid) to prevent damage to water-sensitive clay-rich materials during preparation of samples (by cutting, grinding, and polishing) for observing the surfaces of rock-slabs and thin-sections. All of the surfaces for observation and analysis were prepared to be perpendicular to the fault surface and parallel to the slip direction. To analyze the chemical composition and microstructure of clast and matrix materials in slip zones, we used a scanning electron microscope (SEM) with back-scattered electron (BSE) mode, equipped with an energy dispersive X-ray spectrometer (EDS). The operating acceleration voltage and beam current were 15–20 kV and 1–5 nA, respectively.

To calculate the average stress state from the fault-slip data obtained at outcrops and excavation sites (Table SI1), we used the stress tensor inversion method of Michael^42,43^. The inversion determines the orientation of the three principal stress axes (σ_1_, σ_2_, and σ_3_) and the relative magnitude of the principal stresses [R = (σ_2_ − σ_3_)/(σ_1_ − σ_3_)]. We also applied a bootstrap resampling technique to calculate confidence regions for the stress tensor by assuming that 50% of the planes are picked incorrectly. We followed the procedure of the method in Imanishi et al.^44^.

## Results

### Distribution, geometry, and kinematics of ruptures

**Figure 2:**
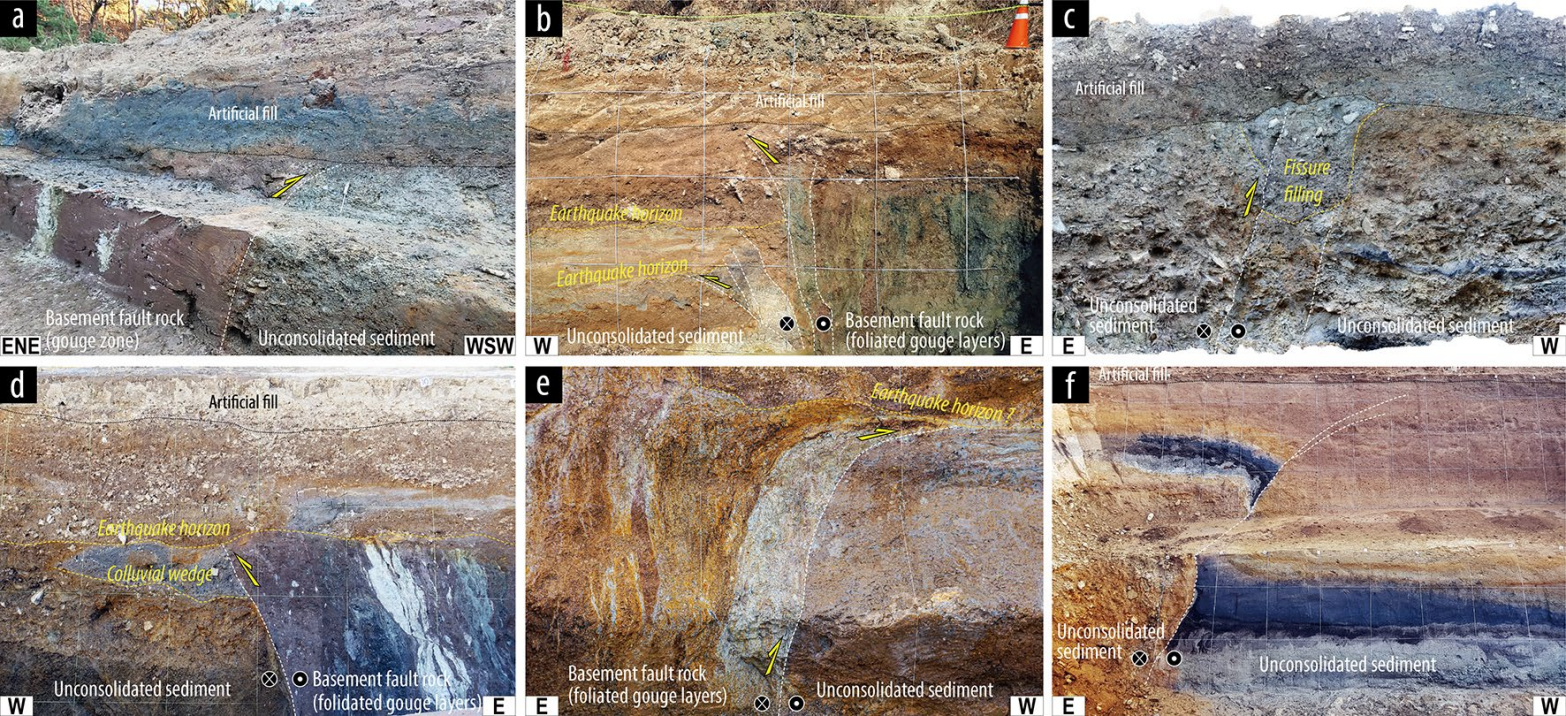
Photographs of paleoearthquake ruptures at several of the studied trench sites along the main fault core and a subsidiary fault of the Yangsan Fault; the (**a**) Geosi, (**b**) Byeokgye, (**c**) Wolsan, and (**d**) Miho along the main fault core and (**e**) Inbo2 and (**f**) Inbo1 sites on a subsidiary fault, from north to south. Dashed white lines indicate the principal slip zones (PSZs).

Paleoearthquake rupture splays observed at the seven trench localities occurred primarily along the main fault strand (or fault core) and subsidiary faults distributed in the fault damage zone (Fig. 2 and Table 1). These rupture splays strike mostly NNE–SSW and dip toward the east at high angle (> 75°) (Tables 1 and SI1), which depend on the pre-existing geometry of the older structures (N10–20°E/ > 75°SE). Logistical constraints were encountered during our survey of the entire fault zone, which has a core of up to 100 m in width and a damage zone measuring several kilometers wide. However, in the case of rupture occurrence along the main fault core (Fig. 2a–d), the rupture splays propagated mainly along a particular boundary between the fault core and the adjoining damage zone, with different material strengths on each side of the slip zone. The < 3 cm-wide domain is regarded as the principal slip zone (PSZ) in each case. The narrow PSZs are expressed as ultrafine clay-rich matrix and submillimeter-sized clasts, which appear to have undergone much stronger grain-size reduction compared with the other gouge layers in a fault core zone. The relationship between the rupture splays and beddings in all trench walls shows an east-side-up apparent offset and dragging of recent unconsolidated strata, indicating a reverse component of movement (Fig. 2). Striations observed on rupture surfaces indicate predominantly dextral-slip to dextral-reverse oblique-slip senses of movement (Tables 1 and SI1). The rupture splays across unconsolidated layers in some cases show that the vertical offset reduces upward. Offset strata display local variation in thickness and sedimentary facies differences across the rupture splays. These features might be controlled by strike-slip rupturing but also by scarp growth resulting from multiple faulting events^18^. Furthermore, several geomorphic features (deflected streams and offset terraces and alluvial fans) show dextral offset from recent paleoearthquakes along some sections of the Yangsan Fault where the trench sites are located^24–28^. Therefore, the general rupture kinematics of the fault under the neotectonic regime can be defined as dextral-slip with a minor reverse component (top-to-the west sense).

### Branched and terminated ruptures in unconsolidated strata

**Figure 3:**
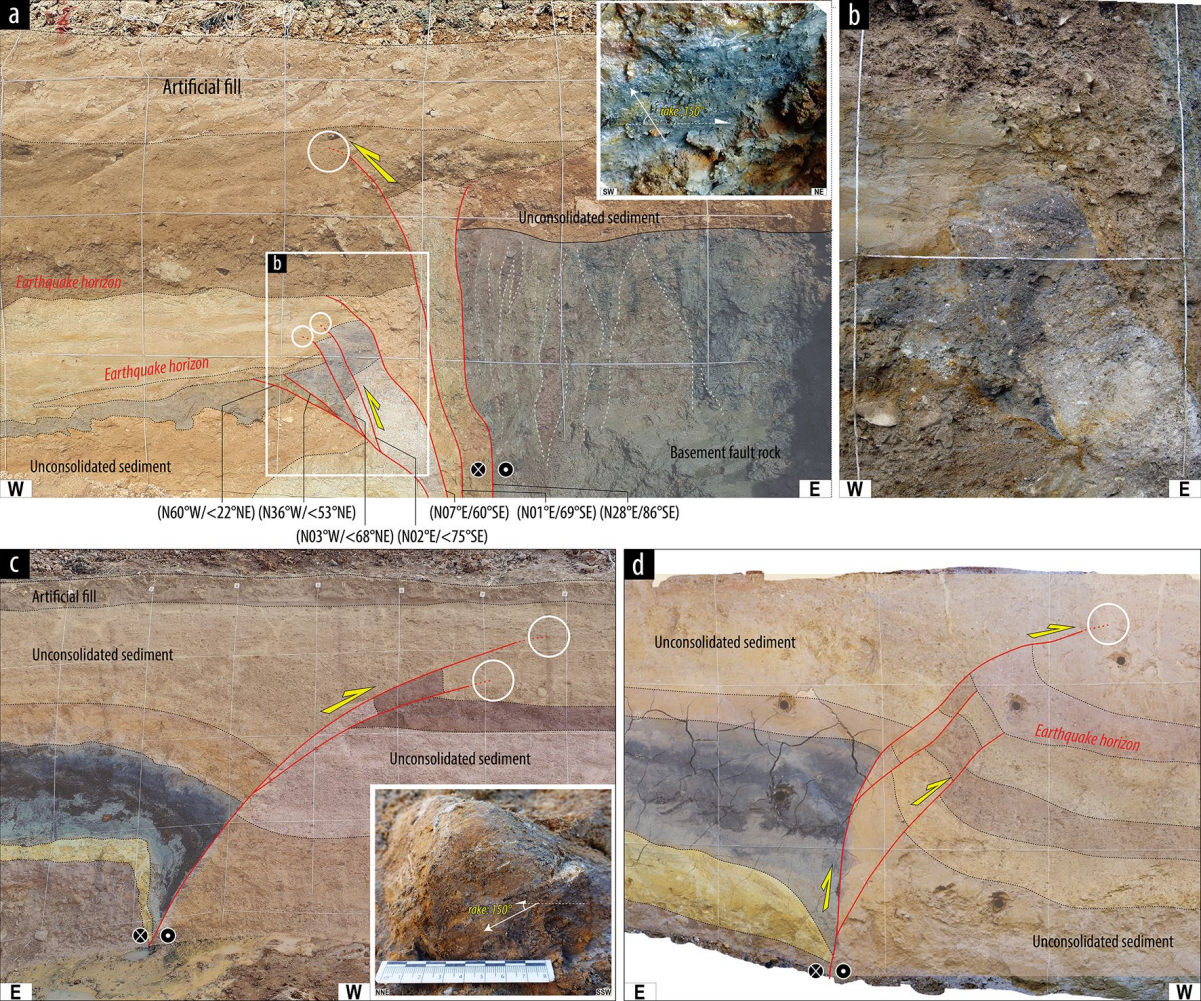
Photographs of paleoearthquake ruptures showing a westward bifurcation and change in geometry and kinematics close to the surface at Byeokgye and Inbo1 trench sites. (**a**) A photograph at Byeokgye site on the main fault core (modified from Song et al.^26^) and (**b**) a close-up view of fault splays. Note that the fault splays have N–S to NW–SE-strikes with low-angle dips in the unconsolidated strata. (**c,d**) Photographs at Inbo1 site on the subsidiary fault (modified from Cheon et al.^28^). The first small trench (**d**) was extended to the ~ 8 m deep, ~ 20 m long deep trench (**c**). White circles indicate the rupture termination locations. Insets in (**a**) and (**c**) show that fault striations observed in the unconsolidated strata. These striations predominantly show dextral-slip with a component of reverse movement. Straight arrows and dashed line indicate the movement direction of the missing blocks and the strike of the fault surfaces, respectively.

The ruptures, which originate from the deep seismogenic zone, have propagated through the basement rocks to overlying unconsolidated Quaternary strata in near-surface region. In the basement rocks, the rupture propagations are restricted to a specific weak zone (a < 3 cm wide PSZ) in the pre-existing fault zone (Figs. 2 and 3). However, in the unconsolidated strata in shallow subsurface, the ruptures tend to branch into several narrower splays (less than a few millimeters wide; Fig. 3). The rupture splays can be divided into (1) those breaking the surface and (2) those terminating below the surface. Surface depositional features distributed along rupture splays, such as fissure fills and colluvial wedges, indicate ruptures that extend to the paleo surface (at the Wolsan and Miho sites; Fig. 2b,c; Table 1). In contrast, for ruptures terminating in shallow subsurface, the branched ruptures in the sediment are expressed in the form of a westward bifurcation geometry (Byeokgye and Inbo1 sites; Fig. 3; Table 1). The dip angles of these branched ruptures decrease upward, and their strikes change from NNE–SSW to NNW–SSE (Fig. 3). Furthermore, some slickensides are observed on rupture surfaces, and the rake angles of striations indicate dextral-reverse oblique slip (see insets in Fig. 3a,c), unlike the mostly strike-slip sense on the high-angle fault splays in the basement rocks. These multi-pronged splays in unconsolidated strata could have been produced by a single faulting event or also by several faulting events, as inferred from stratigraphic features such as earthquake horizons; i.e., the boundary between strata fully offset by rupture and the overlying strata^26–28^.

### Deformation mechanism

Generally, primary on-fault paleoseismic features along the fault—such as surface faulting-induced sedimentation (e.g., fissure fill and colluvial wedge) and faulted and folded stratigraphic units—indicate seismic slip, because creep, slow slip, or small slip during small to moderate earthquakes generally leave no such signs of observable deformation^17^. In addition, recently reported microscopic features in the PSZ of the Yangsan Fault, such as narrow (a few millimeters) micro-slip zones, sharp surfaces, ultrafine (less than a few micrometers) materials, gouge injections, cortex structures, and spalling fractures of clasts, strongly support the occurrence of rapid seismic slip reaching the surface^25,28,35,45^. Therefore, the thin and straight slip zones that are located mostly along the boundary of the wide inherited weak zone have acted as conduits for repeated earthquake rupture propagations and could also be pathways for future rupture propagation when large slip is initiated along this fault at seismogenic depths^35^.

**Figure 4:**
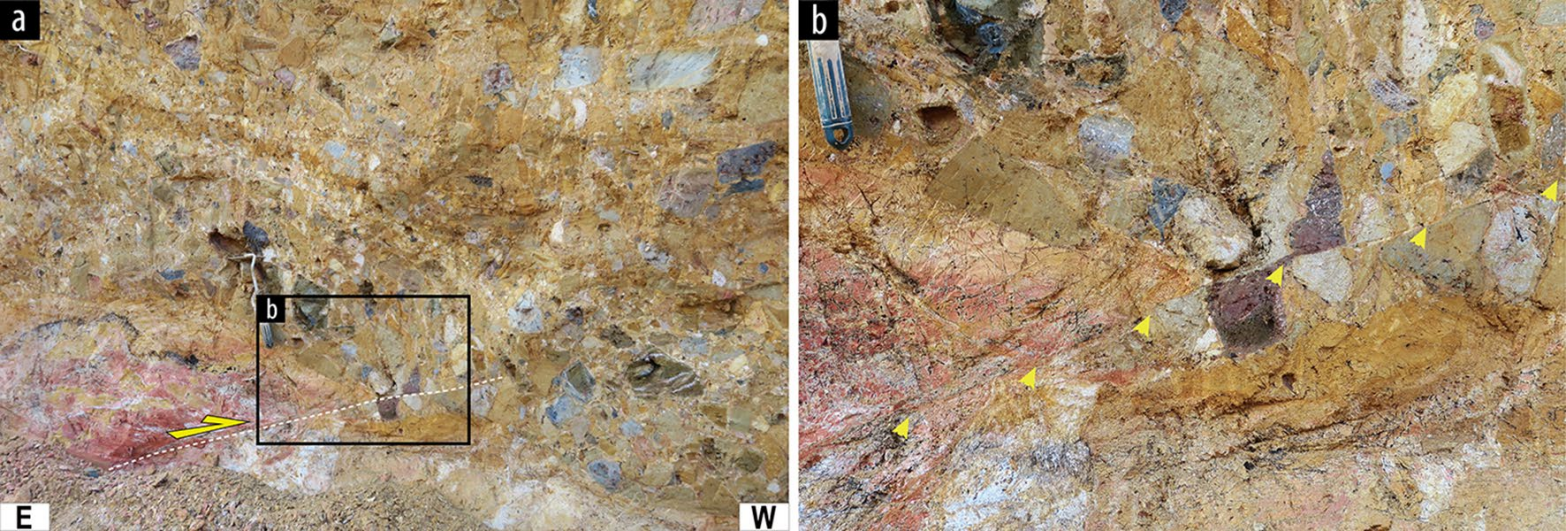
Highly weathered gravels offset by a surface rupture splay observed on the southern YF. There is no strength difference between the clasts and matrix.

**Figure 5:**
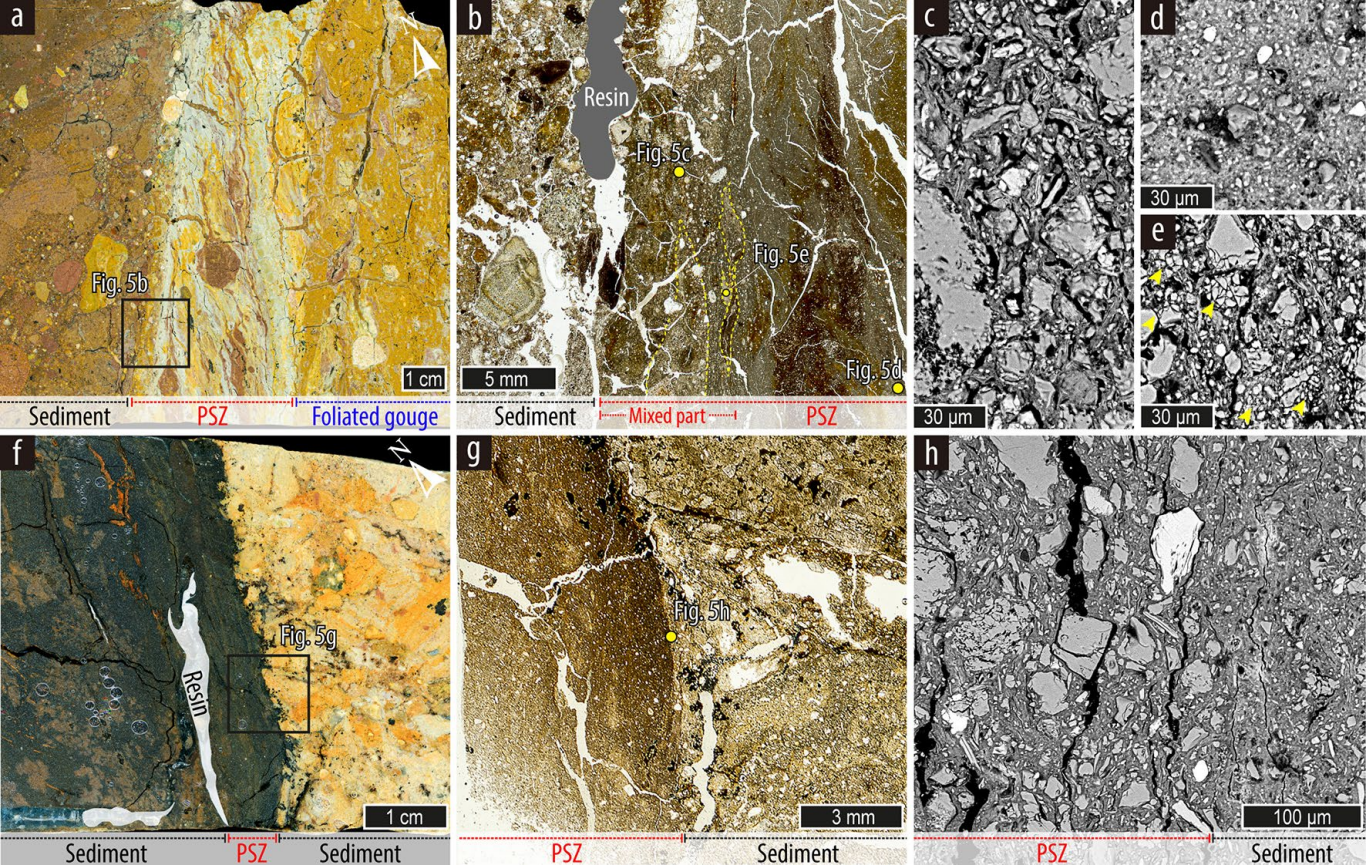
Microstructures of rupture splays at the Inbo2 (**a**–**e**) and Inbo1 (**f**–**h**) sites. (**a**,**b**) Slab (**a**) and thin-section (plane-polarized light) (**b**) images showing a rupture splay along the boundary between mature foliated gouge and unconsolidated gravel. Yellow dashed lines indicate asymmetric lenses derived from sediments and gouges. (**c**,**d**) SEM–BSE images showing thin undulated foliated layers in the mixed part of PSZ (c) and well-rounded clasts in the PSZ (**d**). (**e**) SEM–BSE image showing asymmetric lenses in the mixed part in the PSZ. Yellow arrows depict cataclasis of clasts. (**f**–**h**) Slab (**f**), thin-section (**g**), and SEM–BSE (**h**) images showing a rupture splay along the boundary between dark clay and yellowish sand.

In naturally exposed outcrops or trench walls, rupture splays crossing the non-lithified deposits have generally caused a rearrangement of clasts^28^ rather than transecting them. The larger the size of gravel in the strata, the harder it is to trace a rupture splay. However, at some sites (not presented in Table 1), we found offset gravels in the strata (Fig. 4), revealing a small strength contrast between clasts and matrix because of intense weathering. Microscopic observations of PSZs in unconsolidated sediment at the Inbo2 and Inbo1 sites also reveal granular flow deformation. A rock-slab of a sample collected at the boundary between unconsolidated gravel and old fault rock (foliated gouge) at Inbo2 site shows the < 3-cm-wide PSZ (Fig. 5a,b). The pale-yellow clay-rich PSZ, distinguished from dark-brown gravel sediments and light-brown clay-rich foliated gouges, has a linear geometry and contains asymmetric clasts and S–C fabrics of clay foliations that indicate dextral-slip. In the scanned thin-section image with plane-polarized light, the boundary between gravel and PSZ displays a < 8-mm-wide mixed zone of materials derived from coarse sediment and fine-grained gouge on the two sides, respectively (Fig. 5b). SEM–BSE images of the sediments–gouge mixed zone of PSZ show the clasts surrounded by undulated foliations of platy clay minerals without fragmentation of clasts (Fig. 5c). These features can be observed at shallow depths in various tectonic environments (< 500–1000 m)^46^. Furthermore, fine-grained (< 20 μm in diameter) clasts and micro-sized clay minerals in PSZ show granular flow deformation by reworking of older gouge materials (Fig. 5d), which indicate that the gouges formed by comminution and cataclasis at deep part were reactivated at shallow depth during recent rupture events. However, cataclasis of quartz and feldspar grains, which can be generated from high strain deformation at large depths and coseismic slip propagation to the shallow subsurface^47^, is also observed in asymmetrical lenses (Fig. 5e). Similarly, a boundary between unconsolidated clay and sand at Inbo1 site also shows a < 1-cm-wide PSZ with thin (< 1 mm wide) foliations, which are parallel to the Y-shear direction (Fig. 5f,g). Scanned rock-slab (Fig. 5f) and thin-section (Fig. 5g) images show that the boundary between the PSZ and sand has a straight and sharp contact. However, SEM–BSE image of the boundary displays undulated foliations of clay minerals through-going between clasts without intense cataclasis, such as fragmentation and truncation of clasts (Fig. 5h).

## Discussion

The combined results of our structural observations and previously reported paleoseismic data for the Yangsan Fault show that multiple ruptures propagated repeatedly along a narrow pre-existing weak zone at basement depths and terminated in zones of distributed deformation in unconsolidated strata in the shallow subsurface showing a change in geometry and kinematics. Here, we examine the controls on the inhibition of the upward rupture propagation in the shallow subsurface along the fault.

**Figure 6:**
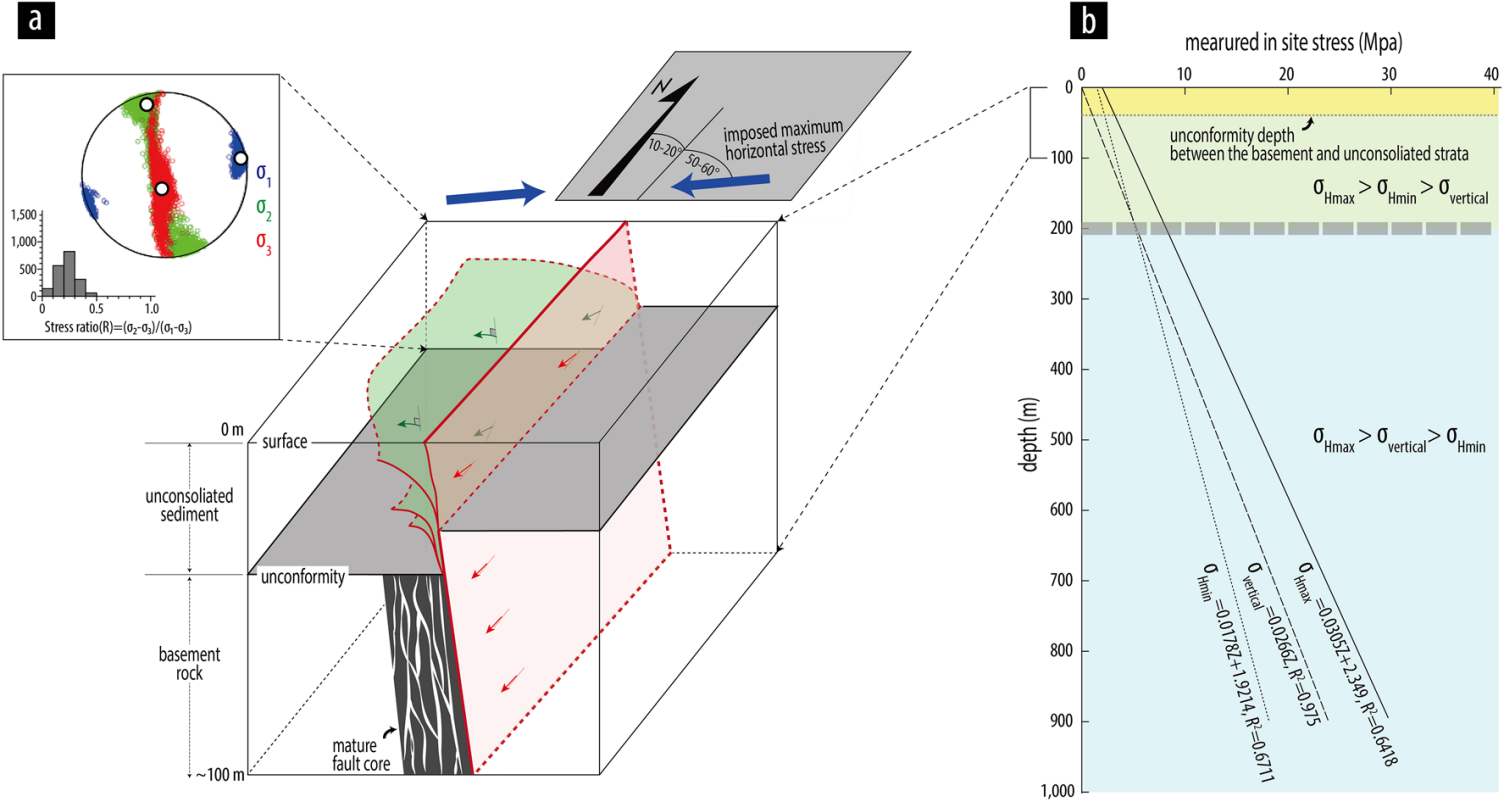
(**a**) Schematic diagram showing the rupture termination process in shallow subsurface along the Yangsan Fault. The stress tensor inversion calculated from fault-slip data (Table SI1) for the fault shows principal stress axes with their 95% confidence regions plotted on lower hemisphere stereonet. (**b**) In situ stress for South Korea measured by hydraulic fracturing and overcoring data (Modified from Kim et al.^48^).

We first consider the role of inherited fault geometry and horizontal tectonic stress, which show an unfavorable relationship in terms of generating slip on the fault. The neotectonic setting of the Korean Peninsula is defined as a strike-slip regime (σ_2_ is σ_v_) with N70 to 75°E of σ_Hmax_, according to focal mechanism data^31–33^. The horizontal angle between the strike of the fault (N10–20°E) and imposed tectonic stress is 50–65°, which is a misfit angle compared with the favorable range of 25–30° proposed by Sibson^12^ (Fig. 6a). Under this unfavorably oriented stress field (maximum horizontal stress of N70–75°E, σ_2_ is σ_v_), the propagation of ruptures is mostly restricted to along a specific pre-existing narrow PSZ (N10–20°E/> 75°SE) with a predominantly dextral-slip sense in basement rocks. In other words, ruptures in the deeper parts are forced to follow the pre-existing weak zone. Interestingly, the PSZ branches into several new splays in unconsolidated strata in the shallow subsurface, whose attitudes tend to be reoriented with a N–S to NNW–SSW strike and an eastward dip of low-angle (Fig. 6a). In addition, primarily oblique-slip kinematics are observed on the N–S to NNW–SSW-striking rupture splay surfaces. We emphasize that an absence of inherited fault enables the formation of new multi-pronged splays in recent sediments that are influenced by the subsurface stress field. In particular, the westward bifurcation geometry is also controlled by the pre-existing dip direction of the inherited structure.

Here, on the basis of the changing geometry and kinematics of the ruptures, we suggest another controlling factor as the depth-dependent change in stress from σ_Hmax_ > σ_v_ > σ_Hmin_ to σ_Hmax_ > σ_Hmin_ > σ_v_ close to the surface (Fig. 6a). Our stress tensor inversion calculated from compiled fault-slip data from shallow depths (outcrops and excavation sites; Table SI1) reveals a transpressive to compressive stress regime in the Korean Peninsula (σ_3_ is σ_v_; Fig. 6a), unlike the stress reconstructions from focal mechanism data (strike-slip stress regime, σ_2_ is σ_v_)^31–33^. Furthermore, a depth–stress relationship in South Korea established by a comprehensive analysis of data for in situ stress measurements using hydraulic fracturing and overcoring methods^48^ has shown that relative magnitudes of σ_v_ and σ_Hmin_ change at a depth of ~ 200 m (Fig. 6b). Another study on the in situ stress field in South Korea, based on borehole hydraulic fracturing tests, has also revealed that the prevailing stress regime changes with depth from reverse faulting (0–200 m depth), to transitional region (200–500 m), and strike-slip faulting (below 500 m)^49^. This vertical change in stress condition can be interpreted in terms of the greater decrease in vertical stress due to the greater release of overburden loading compared with the decrease in σ_Hmin_ closer to the surface. Similarly, Haimson and Voight^50^ reported an example of depth-dependent change in stress from extensional stress regime to strike-slip stress regime at a depth of ~ 250 m in Reykjavik, Iceland. Ma^1^ demonstrated that decreasing overburden pressure plays an important role in the pronounced broadening of damage distribution in near-surface regions based on three-dimensional numerical simulation of dynamic rupture process.

The unconsolidated state of near-surface sediments is another secondary control on rupture termination. The near-surface ruptures across the sediments generally rearranged clasts by rolling and frictional sliding along the boundary of clasts, but clast breakage (cataclasis) also occurs, as revealed by our observations (Figs. 4 and 5) and those of previous studies^35^. Rupture splays are difficult to trace where the sediment is composed of matrix-supported gravels with a considerable strength contrast between matrix and clast. Furthermore, observing micro-scale cataclasis caused by ruptures in fine-grain sediment is also challenging. This deformation mechanism is defined as granular flow and distributed deformation along incohesive inter-granule parts and is a dominant behavior when a fault cuts through non-lithified sediment under low confining pressure^46,47,51,52^. At a much larger scale, based on an analysis of surface deformation patterns in the 2013 M_w_ 7.7 Balochistan, Pakistan earthquake, Zinke et al.^7^ showed that near-surface deformation in younger or thicker non-lithified sediments tends to be more distributed than deformation in basement rocks and old lithified sediments. In addition, image correlation studies of surface deformation patterns of the 1992 M_w_ 7.3 Landers and 1999 M_w_ 7.1 Hector Mine earthquakes suggested that a wider zone of distributed shear occurs where ruptures propagated through loose, unconsolidated sediments^53,54^. These features indicate that the rupture propagation process is influenced by the physical properties and thickness of unconsolidated sediment, which lack inherited structures to constrain distribution of the newly formed ruptures and have low inter-granular cohesion, favoring distributed deformation. Nevertheless, we emphasize that if the sediment matrix and clasts have similar strength because of weathering and consolidation, cataclasis and localized deformation could occasionally occur in the unconsolidated strata during slip events.

We conclude that the near-surface regions on the Yangsan Fault in SE Korea, where are characterized by unconsolidated strata with no pre-existing structures, favor the division of each rupture into several new splays having an orientation with the most favorable attitude with respect to the stress field; i.e., a reverse-slip sense, in response to the imposed stress (ENE–WSW-trending σ_Hmax_, σ_3_ is σ_v_).

## Conclusion

The Yangsan Fault is a typical low-deformation-rate fault in a weakly-active tectonic regime. However, the occurrence of contractional strike-slip paleoearthquake ruptures in near-surface regions indicates a process that inhibits the upward propagation of ruptures. This is apparent as a change from dextral slip on localized pre-existing rupture geometry at basement depths to oblique slip on westward bifurcation rupture geometry in shallow regions. The process is controlled by the following factors: (1) the unfavorable orientation of the inherited fault at basement depths with respect to the tectonic stress field and the lack of upward extension of the inherited fault to the shallow subsurface; (2) the ratio of overburden stress to minimum horizontal stress, which changes from deep to shallow levels (i.e., at ~ 200 m depth); and (3) the physical properties of unconsolidated sediments, which have low inter-granular cohesion that favors granular flow and distributed deformation.

## Supplementary Information

Supplementary Information.

## Data Availability

The datasets used and/or analysed during the current study available from the corresponding author on reasonable request.
