# Supplementary material for: Near-surface termination of upward-propagating strike-slip ruptures on the Yangsan Fault, Korea
Source: Sci Rep. 2023 Jun 19;13:9869. doi: 10.1038/s41598-023-37055-7 (PMC10279704; doi:10.1038/s41598-023-37055-7)
Supplement: Supplementary file 1 — Supplementary Information. [file 41598_2023_37055_MOESM1_ESM.docx]

# **Supplemental Information for:**

Near-surface termination of upward-propagating strike-slip ruptures on the Yangsan Fault, Korea

# **Youngbeom Cheon, Chang-Min Kim*****, Jin-Hyuck Choi, Sangmin Ha, Seongjun Lee, Taehyung Kim, Hee-Cheol Kang, and Moon Son**

[*cmkim247@kigam.re.kr](mailto:*cmkim247@kigam.re.kr)

# **This file includes:**

**Table SI1**

**Table SI1.** Information on slip data on the active fault surfaces at each site along the Yangsan Fault reported by previous studies. Rake is described as the direction of fault motion with respect to the strike, measured anticlockwise from the horizontal line. Values are between -180° and 180° (left-lateral strike-slip: near 0°; right-lateral strike-slip: near 180°; normal-slip: near -90°; reverse-slip: near 90°).

| No. | Site name | Strike | Dip | Rake | Kinematics | Location (Latitude, Longitude) | Reference |
| --- | --- | --- | --- | --- | --- | --- | --- |
| 1 | Gusan | 030 | 55 | 166 | RL+R | 36.942544 129.359217 | Jin et al. [1] |
| 2 | Gusan | 028 | 60 | 142 | RL+R | 36.942544 129.359217 | Choi et al. [2] |
| 3 | Pyeonghae | 000 | 90 | -170 | RL+N | 36.747111 129.444333 | Choi et al. [2] |
| 4 | Jabuteo | 008 | 80 | -160 | RL+N | 36.445583 129.401944 | Yang [3] |
| 5 | Jabuteo | 008 | 89 | -160 | RL+N | 36.201361 129.304667 | Choi et al. [2] |
| 6 | Yugye | 000 | 45 | 140 | RL+R | 36.201361 129.304667 | Kyung [4] |
| 7 | Yugye | 015 | 42 | 102 | RL+R | 36.201361 129.304667 | Kyung and Chang [5] |
| 8 | Yugye | 000 | 45 | 103 | RL+R | 36.201361 129.304667 | Kyung and Chang [5] |
| 9 | Yugye | 013 | 45 | 111 | RL+R | 36.201361 129.304667 | Choi et al. [2] |
| 10 | Yugye | 026 | 46 | 125 | RL+R | 36.201361 129.304667 | Park et al. [6] |
| 11 | Yugye | 017 | 44 | 102 | RL+R | 36.201361 129.304667 | Kyung et al. [7] |
| 12 | Bangok | 225 | 85 | 155 | RL+R | 36.170250 129.282889 | Park et al. [6] |
| 13 | Bangok | 222 | 85 | 154 | RL+R | 36.170250 129.282889 | Choi et al. [2] |
| 14 | Byeokgye (outcrop) | 355 | 80 | 180 | RL | 36.059889 129.270139 | Park et al. [41] |
| 15 | Byeokgye (outcrop) | 011 | 80 | 172 | RL+R | 36.059889 129.270139 | Choi et al. [2] |
| 16 | Byeokgye | 014 | 72 | 150 | RL+R | 36.069080 129.255520 | Song et al. [8] |
| 17 | Dangu | 012 | 79 | 165 | RL+R | 36.059639 129.253889 | Lee et al. [9] |
| 18 | Inbo1 | 017 | 75 | 150 | RL+R | 35.642630 129.164370 | Cheon et al. [10] |
| 19 | Sangcheon1 | 020 | 75 | 155 | RL+R | 35.535164 129.108322 | Choi et al. [2] |
| 20 | Sangcheon2 | 015 | 85 | 135 | RL+R | 35.534361 129.111961 | Park et al. [6] |
| 21 | Sangcheon2 | 020 | 75 | 155 | RL+R | 35.534361 129.111961 | Kyung et al. [7] |
| 22 | Sangcheon2 | 025 | 80 | 157 | RL+R | 35.534361 129.111961 | Kyung [4] |
| 23 | Gacheon1 | 004 | 82 | 83 | LL+R | 35.520556 129.101778 | Park et al. [6] |
| 24 | Gacheon1 | 015 | 75 | 167 | RL+R | 35.520556 129.101778 | Choi et al. [2] |
| 25 | Gacheon1 | 026 | 70 | 169 | RL+R | 35.520556 129.101778 | Choi et al. [2] |
| 26 | Gacheon1 | 014 | 80 | 157 | RL+R | 35.520556 129.101778 | Choi et al. [2] |
| 27 | Gacheon2 | 030 | 84 | 161 | RL+R | 35.521306 129.106639 | Park et al. [6] |
| 28 | Gacheon3 | 004 | 85 | 86 | LL+R | 35.521306 129.106639 | Park et al. [6] |
| 29 | Joil | 042 | 82 | 161 | RL+R | 35.505917 129.099833 | Park et al. [6] |
| 30 | Wolpyeong1 | 020 | 90 | 160 | RL+R | 35.497481 129.091217 | Choi et al. [2] |
| 31 | Wolpyeong2 | 014 | 49 | 122 | RL+R | 35.496275 129.093092 | Choi et al. [2] |

# **References**

1. Jin, K., Kim, Y. -S., Kang, H. C., & Shin H. C., Study on developing characteristics of the Quaternary Gusan Fault in Uljin, Gyeongbuk, Korea: J. Geol. Soc. Korea, **49**, 197‒207 (2013).
2. Choi, S. J., Jeon, J. S., Song, K. Y., Kim, H. C., Kim, Y. H., Choi, P. Y., Choi, W. C., Han, J. G., Ryoo, C. R., Sun, C. G., Jun, M. S., Kim, G. Y., Kim, Y. B., Lee, H. J., Shin, J. S., Lee, Y. S., Gi, W. S., Lee, H. K., Song, Y. G., Kim, Y. S., Kang, T. S., Hong, D. G., & Kim, S. K., Active Fault Map and Seismic Hazard Map: Korea Institute of Geoscience and Mineral Resources, <https://doi.org/10.23000/TRKO201600010825> (2012).
3. Yang, J. S., Quaternary fault activity in the southeastern part of the Korean Peninsula [Ph.D. thesis]: Chuncheon, Kangwon National University, 382 p (in Korean with English abstract) (2006).
4. Kyung, J. B., Trench survey on the neotectonic fault activity on the Sangcheon-ri area of the southern Yangsan Fault zone, in Proceedings, KSEEG Conference: Seoul, Apr 2002, 293‒297 (in Korean) (2002).
5. Kyung, J. B., & Chang, T. W., 2001, The Lastest Fault Movement on the Northern Yangsan Fault Zone around the Yugye-Ri Area, Southeast Korea: J. Geol. Soc. Korea, **37**, 563‒577 (in Korean with English abstract).
6. Park, Y., Ree, J. -H., & Yoo, S. -H., 2006, Fault slip analysis of Quaternary faults in southeastern Korea: Gondwana Research, **9**, 118‒125, <https://doi.org/10.1016/j.gr.2005.06.007>
7. Kyung, J. -B., Lee, K., & Okada, A., A Paleoseismological Study of the Yangsan Fault-Analysis of Deformed Topography and Trench Survey: J. Geol. Soc. Korea, **2**, 155‒168 (in Korean with English abstract) (1999).
8. Song, Y., Ha, S., Lee, S., Kang, H. -C., Choi, J. -H., & Son, M., Quaternary structural characteristics and paleoseismic interpretation of the Yangsan Fault at Dangu-ri, Gyeongju-si, SE Korea, through trench survey: J. Geol. Soc. Korea, **56**, 155‒173 (in Korean with English abstract), DOI: <http://dx.doi.org/10.14770/jgsk.2020.56.2.155> (2020).
9. Lee, J., Rezaei, S., Hong, Y., Choi, J. -H., Choi, J. -H., Choi, W. -H., Rhee, K. -W., & Kim, Y. -S., Quaternary fault analysis through a trench investigation on the northern extension of the Yangsan fault at Dangu-ri, Gyungju-si, Gyeongsangbuk-do: J. Geol. Soc. Korea, **51**, 471‒485 (in Korean with English abstract) (2015).
10. Cheon, Y., Choi, J. -H., Kim, N., Lee, H., Choi, I., Bae, H., Rockwell, T. K., Lee, S. R., Ryoo, C. -R., Choi, H., & Lee, T. -H., Late Quaternary transpressional earthquakes on a long-lived intraplate fault: A case study of the Southern Yangsan Fault, SE Korea: Quaternary International, **553**, 132‒143, DOI: <https://doi.org/10.1016/j.quaint.2020.07.025> (2020).
